# COMMD1-Mediated Ubiquitination Regulates CFTR Trafficking

**DOI:** 10.1371/journal.pone.0018334

**Published:** 2011-03-31

**Authors:** Loïc Drévillon, Gaëlle Tanguy, Alexandre Hinzpeter, Nicole Arous, Alix de Becdelièvre, Abdel Aissat, Agathe Tarze, Michel Goossens, Pascale Fanen

**Affiliations:** 1 INSERM, Unité U955, Créteil, France; 2 Université Paris-Est, Faculté de Médecine, UMR-S 955, Créteil, France; 3 AP-HP, Groupe Henri-Mondor Albert-Chenevier, Service de Biochimie-Génétique, Créteil, France; Ludwig-Maximilians-Universität München, Germany

## Abstract

The CFTR (cystic fibrosis transmembrane conductance regulator) protein is a large polytopic protein whose biogenesis is inefficient. To better understand the regulation of CFTR processing and trafficking, we conducted a genetic screen that identified COMMD1 as a new CFTR partner. COMMD1 is a protein associated with multiple cellular pathways, including the regulation of hepatic copper excretion, sodium uptake through interaction with ENaC (epithelial sodium channel) and NF-kappaB signaling. In this study, we show that COMMD1 interacts with CFTR in cells expressing both proteins endogenously. This interaction promotes CFTR cell surface expression as assessed by biotinylation experiments in heterologously expressing cells through regulation of CFTR ubiquitination. In summary, our data demonstrate that CFTR is protected from ubiquitination by COMMD1, which sustains CFTR expression at the plasma membrane. Thus, increasing COMMD1 expression may provide an approach to simultaneously inhibit ENaC absorption and enhance CFTR trafficking, two major issues in cystic fibrosis.

## Introduction

The cystic fibrosis transmembrane conductance regulator (CFTR) is a cAMP-regulated Cl^-^ channel encoded by the gene mutated in cystic fibrosis (CF) [1]. CF is the most common severe autosomal recessive genetic disorder in Caucasians. The lack of CFTR function at the apical membrane of epithelial cells is the cause of the morbidity and mortality associated with the disease [2]. CFTR is a 1480 amino acid glycoprotein predicted to consist of two membrane-spanning domains, each containing six transmembrane domains (TMD), two cytoplasmic nucleotide-binding domains, a regulatory region and four intracytoplasmic loops (ICLs) connecting the TMDs on the cytoplasmic side of the protein [1]. CFTR is a large polytopic protein whose biogenesis is inefficient and slow, with 60–80% of CFTR being degraded in the endoplasmic reticulum (ER) [3], [4]. It is the first integral membrane protein shown to be a substrate for ER-associated degradation (ERAD) *via* the ubiquitin proteasome system. Proteasomal degradation occurs in both the wild-type CFTR (wt-CFTR) and the disease-associated F508del mutant [5], [6]. Ubiquitination can also regulate CFTR at the plasma membrane and internalized CFTR can either be ubiquitinated and diverted for lysosomal degradation or can be recycled back to the cell surface [7]–[10]. However, identifying new regulators of CFTR membrane trafficking in post-Golgi compartments is still a major research issue.

COMMD1, previously known as MURR1 (Mouse U2af1-rs1 region 1), is the prototype of a new family of 10 proteins containing COMM (*Copper Metabolism gene MURR1*) domains, from COMMD1 to COMMD10 [11]. COMMD1 is ubiquitously expressed and was first identified as being involved in the regulation of hepatic copper excretion [12]. A growing body of data suggests that COMMD1 is associated with the ubiquitin proteasome system and regulates the stability of proteins such as NF-κB subunits, ATP7B and HIF-1α [13], [14].

**Figure 1:**
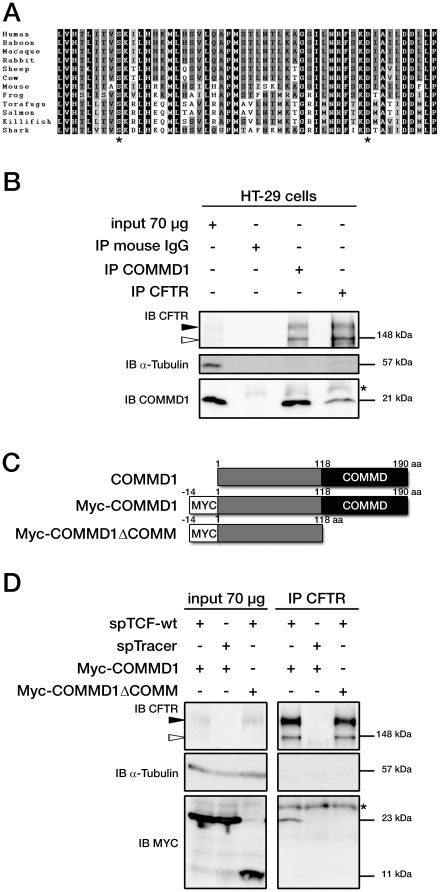
COMMD1 and CFTR interact in mammalian cells. (A) Sequences of ICL3 in other species from fish to primates. Asterisks indicate the position of two class II mutations: S945L and D979A. Identity of amino acids between the different proteins are boxed in black, conserved residues are boxed in dark gray and semi-conserved substitutions in light gray. (B) Representative gels for the same co-immunoprecipitation experiments in HT-29 cells expressing endogenous CFTR and COMMD1. Lysates from HT-29 cells were immunoprecipitated (IP) with either 0.8 µg of anti-COMMD1 mAb (Abnova), 0.8 µg of anti-CFTR mAb (MAB25031, R&D Systems) or with 0.8 µg anti-mouse IgG as a control. Each immunoprecipitation sample was then split in half and loaded onto an 8% SDS-PAGE for CFTR detection and 11% SDS-PAGE for COMMD1 detection. Both gels were transferred to PVDF membrane and subjected to immunoblotting (IB). The 8% SDS-PAGE membrane was probed with anti-CFTR mAb (MM13-4) and the 11% SDS-PAGE membrane with a rabbit anti-COMMD1 pAb (Proteintech Group). Both membranes were probed with anti-α-tubulin as control (11% gel is shown). Filled and empty arrowheads indicate the fully- (170 kDa) and core-glycosylated (140 kDa) CFTR, respectively. * indicates mouse IgG light chain from the antibody used for immunoprecipitation. (C) COMMD1 constructions in pcDNA3.1/Topo plasmid. Two COMMD1 constructs were generated by adding a Myc-tag at the N-terminus of COMMD1: Myc-COMMD1 and a construct with a deletion of the COMM domain named Myc-COMMD1ΔCOMM. (D) Representative gels for the same co-immunoprecipitation experiment between COMMD1 and wt- in heterologous system. HeLa cells stably expressing wt- (spTCF-wt) or empty CFTR vector (spTracer) as control were transfected with Myc-COMMD1. spTCF-wt were transfected with Myc-COMMD1ΔCOMM. Lysates from all these experiments were subjected to SDS-PAGE, as in (B) after CFTR IP. The 8% SDS-PAGE membrane was probed with anti-CFTR mAb and the 11% SDS-PAGE membrane with anti-c-Myc mAb. Both membranes were probed with anti-α-tubulin as control (11% gel is shown).

To better understand the regulation of CFTR processing and trafficking, we performed a yeast two-hybrid screen using the third intracytoplasmic loop (ICL3) of CFTR as bait [15]. Two ICL3 mutations (S945L, D979A) are associated with the CF phenotype (Figure 1A), suggesting the importance of this region in the maturation and/or function of CFTR [16], [17]. COMMD1 was found to exhibit specific and strong binding to ICL3. Our data provide evidence that COMMD1 interacts with CFTR and promotes CFTR cell surface expression through inhibition of CFTR ubiquitination. Regulation of the ubiquitin pathway is one of the basic functions of the COMMD1 protein, which has been shown to contribute to protein degradation [13]. More recently, COMMD1 was also reported to regulate channel cell surface expression, and has been shown to modulate sodium transport in epithelial cells through regulation of ENaC cell surface expression [18]. We suggest that COMMD1 may function as part of the endocytic machinery, helping to regulate the intracellular trafficking process. In addition, COMMD1 may act as a scaffold for protein ubiquitination, thereby affecting protein trafficking.

## Results

### COMMD1 and CFTR interact in epithelial cells

A yeast two-hybrid screen was performed to identify proteins that interact with CFTR amino acids Leu937 to Pro988 (Figure 1A) [15]. One of 36 clones contained a cDNA insert with a near full-length human COMMD1 coding sequence (amino acids 5–190). To assess whether the association of CFTR and COMMD1 had any biological and physiological relevance, we first conducted co-immunoprecipitation experiments with both full-length proteins. We used the human HT-29 colon adenocarcinoma cell line because it expresses both CFTR and COMMD1 endogenously and was previously used for co-immunoprecipitation experiments [15]. CFTR is a 170 kDa glycoprotein whose processing can be assessed by examining its glycosylation status [19]. Electrophoresis of immunoprecipitated wt-CFTR resulted in two close bands (Figure 1B). The first of these was a diffuse band of approximately 170 kDa (band C), representing the mature, fully glycosylated protein processed through the Golgi complex. The second was a thin band of approximately 140 kDa (band B), representing the core-glycosylated protein located in the endoplasmic reticulum. Co-immunoprecipitation experiments performed on HT-29 cells showed an association between COMMD1 and wt-CFTR *in vivo* when using anti-CFTR antibody (Figure 1B). Switching the antibodies used for immunoprecipitation and immunoblotting showed that COMMD1 interacted not only with the core-glycosylated form of CFTR but also with the fully glycosylated form of CFTR, since both bands could be detected clearly on the gel.

COMMD1 is a member of a family defined by the presence of a conserved and unique motif named the COMM domain, which functions as an interface for protein-protein interactions [11]. Therefore, we analyzed the role of this domain in the CFTR-COMMD1 interaction by constructing N-terminal-tagged COMMD1 mammalian vectors (Figure 1C). A full-length construct (Myc-COMMD1) and a COMM domain-truncated construct (Myc-COMMD1ΔCOMM) were transiently transfected in HeLa cells stably expressing wt-CFTR [15]. Co-immunoprecipitation experiments clearly showed that the COMM domain was required for the CFTR-COMMD1 interaction (Figure 1D) and confirmed that both glycosylated forms of CFTR were able to bind to COMMD1.

### COMMD1 regulates CFTR cell surface expression

**Figure 2:**
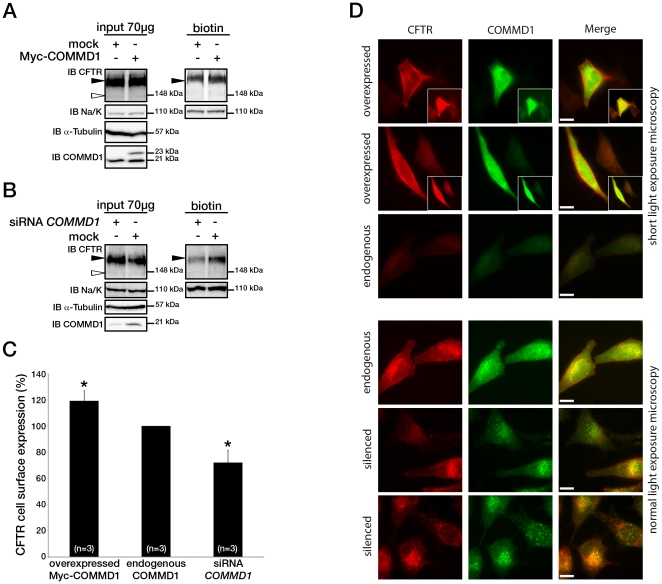
COMMD1 regulates CFTR cell surface expression. (A) HeLa cells stably expressing wt-CFTR were transiently transfected with an empty COMMD1 vector (mock, pcDNA3.1/Topo) or Myc-COMMD1, and were biotinylated with Sulfo-NHS-LC-biotin. Lysates from all these experiments were subjected to SDS-PAGE directly (input) or pulled-down with streptavidin-agarose (biotin). Representative gels for the same samples were separated by 8% SDS-PAGE for CFTR, Na/K-ATPase detection and 11% SDS-PAGE for COMMD1, α-tubulin detection. (B) HeLa cells stably expressing wt-CFTR were transiently transfected with a siCONTROL Non-Targeting siRNA (mock) or COMMD1 siRNA and further processed as in (A). Filled and empty arrowheads indicate the fully- (170 kDa) and core-glycosylated (140 kDa) CFTR, respectively. (C) Quantification of CFTR cell surface expression. The biotinylated CFTR level is normalized to the biotinylated Na/K-ATPase level. Endogenous COMMD1 expression is referred as 100%, with mock being pcDNA3.1/Topo for overexpression experiments (A), whereas mock was siCONTROL for silencing experiments (B). The means ± S.D. were obtained from three independent experiments.* P<0.05 was determined by t-test. (D) Immunofluorescence microscopy of COMMD1 and CFTR in HeLa cells stably expressing wt-CFTR. Cells were transfected with Myc-COMMD1 or COMMD1 siRNA for overexpression and silencing studies, respectively, and not transfected for endogenous expression studies. Two types of light exposure microscopy (short and normal) are shown to visualize all expression conditions. Scale bars: 10 µm.

Studies on the Wilson disease protein showed that COMMD1 participates in the ATP7B-mediated copper-excretion pathway [20]. The exact function of COMMD1 in this pathway remains elusive, but it has been shown to regulate ATP7B trafficking [21]. Furthermore, COMMD1 was recently shown to be involved in ENaC cell surface expression [18]. To determine if COMMD1 participates in CFTR trafficking, we first examined the role of its overexpression on the maturation of the CFTR glycoprotein. Then, we examined CFTR cell surface expression through biotinylation experiments. We observed that a 2-fold overexpression of COMMD1 did not change the amounts of the individual B and C bands, nor did it alter the C/B+C ratio, which indicates that it did not affect CFTR maturation (Figure 2A). However, as shown in Figure 2C, overexpression of COMMD1 increased the cell surface expression of CFTR protein by 20% (119±8%). CFTR expression was normalized to Na/K-ATPase expression. These results were confirmed by immunostaining showing that cells transfected with COMMD1 exhibited an intense plasma membrane staining compared to cells in the same field expressing COMMD1 endogenously (Figure 2D). Taken together, these results indicate that COMMD1 overexpression enhances CFTR cell surface expression.

Loss-of-function studies were conducted using RNA-mediated interference, in which endogenous COMMD1 expression was reduced to 35% of control (Figure 2B). The steady-state level of CFTR was not affected; however, biotinylation experiments clearly showed a 30% decrease in CFTR membrane expression (72±9%) (Figure 2C). Immunostaining confirmed that CFTR expression was strongly reduced at the cell surface even if COMMD1 was not completely silenced (Figure 2D). Therefore, we can conclude that COMMD1 down-regulation inhibits CFTR cell surface expression.

### COMMD1 subcellular distribution

**Figure 3:**
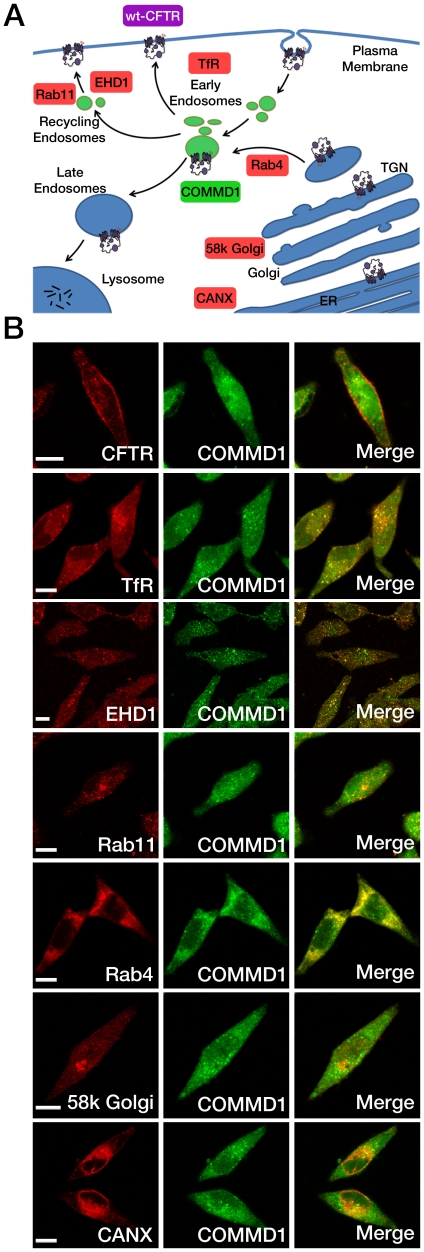
COMMD1 colocalization with organelle markers. (A) Cartoon indicating the location of the different markers (red), CFTR (violet) processing throughout the cell and the intracellular localization of COMMD1 (green). (B) Double-labeling studies with organelle markers were performed in stably expressing wt-CFTR HeLa cells. Immunostaining was performed on fixed and permeabilized cells using rabbit primary antibody (anti-COMMD1 pAb 1∶100) followed by Alexa Fluor 488-conjugated goat anti-rabbit secondary antibody (green), and each organelle marker was labeled with mouse primary antibodies (calnexin 1∶400, 58k Golgi 1∶100, Rab4 1∶100, Rab11 1∶100, EHD1 1∶100, TfR 1∶200) followed by Alexa Fluor 555-conjugated goat anti-mouse secondary antibody (red). Non-immune anti-mouse and anti-rabbit controls were conducted to remove non-specific signals. Fluorescence images of cells were captured and analyzed with a Zeiss Axio Observer Z.1 confocal microscope with x63 objective. Scale bars: 10 µm.

Endogenous COMMD1 subcellular distribution is both nuclear and cytoplasmic, with cytoplasmic expression restricted to vesicular compartments, as observed in HeLa cells [12]. To further define the intracellular localization of COMMD1, double-labeling experiments of COMMD1 and organelle markers were performed in our wt-CFTR expression model (Figure 3). Distribution of COMMD1 did not overlap with the ER marker calnexin, the trans-Golgi network marker 58k or the endosomal marker Rab4. Partial colocalization was observed with the recycling endosome markers Rab11 and EHD1. The best colocalization was observed with the transferrin receptor (TfR), which is a marker of early/recycling endosomes.

### COMMD1 regulates CFTR ubiquitination

**Figure 4:**
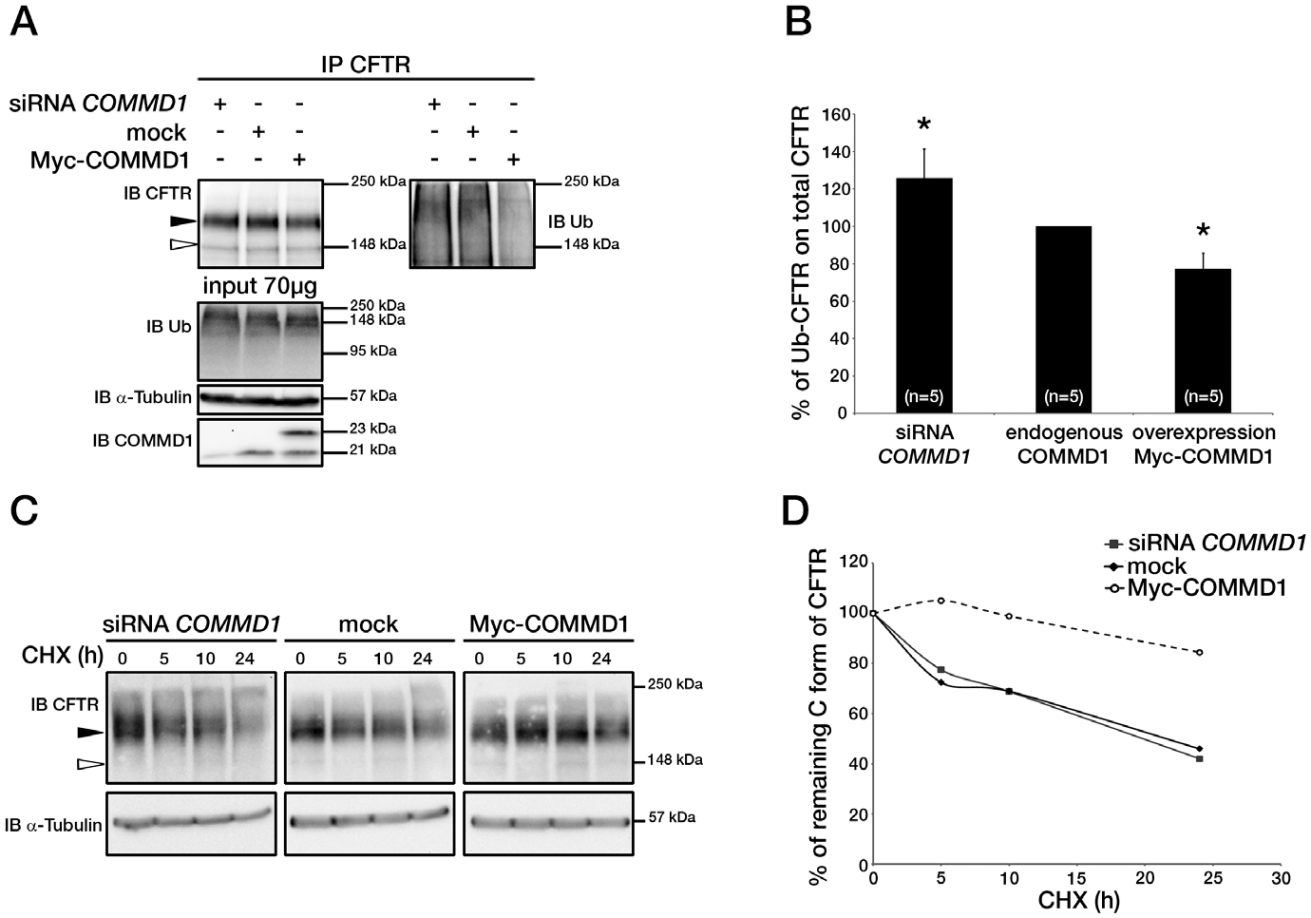
COMMD1 regulates CFTR ubiquitination. (A) Representative gels for the same CFTR IP experiment with MAB25031 from HeLa cells stably expressing wt-CFTR and separated on 8% SDS-PAGE transferred to PVDF membrane. Half of the membrane was probed with anti-CFTR mAb and the other half with anti-ubiquitin mAb. Lysates were loaded onto an 11% SDS-PAGE and sequential probing of the membrane was performed (COMMD1, α-tubulin and lastly ubiquitin). Filled and empty arrowheads indicate the fully- (170 kDa) and core-glycosylated (140 kDa) CFTR, respectively. (B) Quantification of ubiquitinated CFTR. Ratio of ubiquitinated CFTR to total CFTR in each condition is shown, endogenous COMMD1 expression is referred as 100%. The means ± S.D. were obtained from five independent experiments.* P<0.05 was determined by t-test. (C) Stability of the mature wt-CFTR was determined upon inhibition of protein biosynthesis with cycloheximide (CHX). Cells were incubated in the presence of cycloheximide for the indicated time intervals. (D) Quantification of mature CFTR was normalized to α-tubulin level.

Several studies indicate that COMMD1 is a regulator of protein ubiquitination involved in ENaC trafficking and in the HIF-1 and NF-κB pathways [22]–[25]. Within the NF-κB pathway, COMMD1 promotes the ubiquitination of RelA, whereas it protects IκBα from ubiquitination. These studies prompted us to test whether COMMD1 expression affects CFTR ubiquitination in HeLa cells stably expressing wt-CFTR. Immunoprecipitation followed by immunoblotting showed that the overall expression of CFTR did not change when COMMD1 was up- or down-regulated (Figure 4A), which is consistent with results from whole cell lysates (Figure 2A and 2B). The amount of ubiquitinated CFTR was then assessed in the absence of proteasome inhibitors by blotting the same membrane with a monoclonal anti-ubiquitin antibody (Figure 4A, right panel). Densitometric analysis showed that the relative amount of ubiquitinated CFTR normalized to total CFTR was significantly altered with respect to COMMD1 expression (Figure 4B). COMMD1 down-regulation increased ubiquitinated CFTR (126±15%), whereas its overexpression decreased ubiquitinated CFTR (77±8%). No change was observed in total protein ubiquitination. HeLa cells stably expressing wt-CFTR were transiently transfected with Myc-COMMD1, vector alone or COMMD1 siRNA and subsequently incubated with the protein synthesis inhibitor cycloheximide for different time intervals. Immunoblotting of whole cell lysates demonstrated that when COMMD1 was overexpressed, mature CFTR stability was notably increased compared to controls (Figure 4C and 4D). Altogether, these data clearly demonstrate that COMMD1 affects CFTR protein stability and selectively regulates CFTR ubiquitination without affecting the overall ubiquitination process.

### COMMD1 regulates CFTR ubiquitination through an ICL3 motif

**Figure 5:**
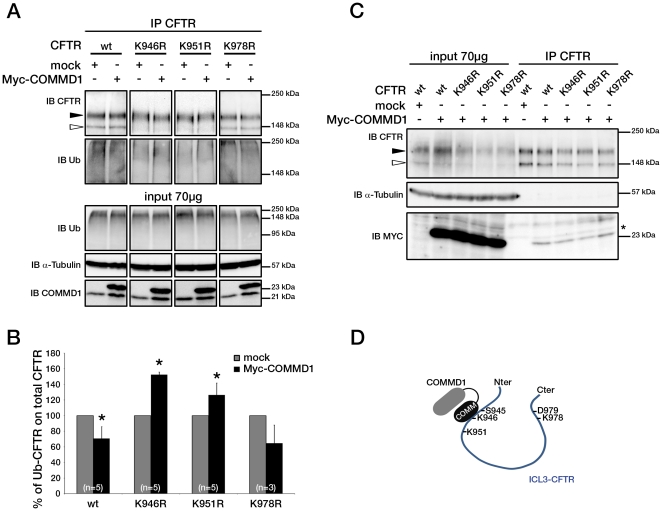
COMMD1 regulates CFTR ubiquitination through an ICL3 motif. (A) HeLa cells were transfected with CFTR constructs (wt, K946R, K951R or K978R-CFTR) and Myc-COMMD1 or empty vector as control (mock). The same quantities of lysates were immunoprecipitated with anti-CFTR mAb (MAB25031). Representative gels for the same experiment where each immunoprecipitation sample was then split in half and loaded onto two 8% SDS-PAGEs for CFTR detection and ubiquitin detection. Both gels were transferred to PVDF membrane and subjected to immunoblotting (IB). Lysates were loaded onto an 11% SDS-PAGE and sequential probing of the membrane was performed (COMMD1, α-tubulin and lastly ubiquitin). Filled and empty arrowheads indicate the fully- (170 kDa) and core-glycosylated (140 kDa) CFTR, respectively. (B) Quantification of ubiquitinated CFTR. Ratio of ubiquitinated CFTR to total CFTR in cells transfected with Myc-COMMD1 compared to the same ratio in mock-transfected cells was reported as 100% for each independent experiment. The means ± S.D. were obtained from five to three independent experiments.* P<0.05 is determined by t-test. (C) Representative gels for the same co-immunoprecipitation experiment between COMMD1 and wt, K946R, K951R or K978R-CFTR in heterologous system. HeLa cells were co-transfected with Myc-COMMD1 or empty vector (mock) and wt, K946R, K951R or K978R-CFTR. Lysates from all these experiments were subjected to SDS-PAGE after CFTR IP. The 8% SDS-PAGE membrane was probed with anti-CFTR mAb and the 11% SDS-PAGE membrane with anti-c-Myc mAb. Both membranes were probed with anti-α-tubulin as control (11% gel is shown). (D) Proposed model of COMMD1 interaction through its COMM domain with the N-terminal end of ICL3 to modulate CFTR ubiquitination.

As part of the yeast two-hybrid screen, targeted two-hybrid tests between COMMD1 and ICL3 mutants S945L and D979A were conducted. A loss of interaction was observed with S945L, suggesting that the substitution of Ser-945 disrupts the ICL3/COMMD1 interaction in yeast in contrast to that of Asp-979 (data not shown). Since our results showed that COMMD1 overexpression inhibited CFTR ubiquitination selectively, we hypothesized that well-conserved lysine residues located in the ICL3 domain could be potential ubiquitin targets. Three lysines were mutated to arginine in the ICL3 domain near Ser-945 (K946R and K951R) and Asp-979 (K978R). Mutant CFTR proteins were transiently transfected in HeLa cells. When COMMD1 was overexpressed, ubiquitination of K946R- and K951R-CFTR was increased (152±3% and 126±15%, respectively) whereas ubiquitination of wt-CFTR was decreased (70±15%) (Figure 5A and 5B). The difference between mock-transfected and COMMD1-transfected cells was not significant in the case of mutant K978R-CFTR. Thus, COMMD1 overexpression led to a decrease in ubiquitinated wt-CFTR in both stably- and transiently-transfected cells. However, ubiquitination of K946R-CFTR and K951R-CFTR was significantly increased compared to that of wt-CFTR (Figure 5B). The co-immunoprecipitation experiments between COMMD1 and each of the three mutant CFTR proteins did not show any loss of interaction between COMMD1 and CFTR (Figure 5C). Altogether, these data clearly demonstrate that the ICL3 CFTR residues located from Ser-945 to Lys-951 are implicated in the interaction with COMMD1 (Figure 5D).

## Discussion

COMMD1 is a recently identified protein that is associated with multiple cellular pathways. Initially described as a protein involved in copper metabolism [26], a growing number of studies have identified COMMD1-associated proteins in sodium transport [27], NF-κB signaling [11], [23], HIF-1 signaling [25], ubiquitination [23], [24] and apoptosis [28]. Thus, COMMD1 is now considered to be a pleiotropic protein with various cellular functions [14].

The present study shows that COMMD1 binds to CFTR and that this interaction occurs with both the immature and mature forms of the CFTR protein in cells expressing endogenous CFTR and COMMD1. Overexpression of COMMD1 led to a 20% increase in CFTR on the cell surface, whereas its down-regulation decreased CFTR cell surface expression by 35%. These results imply that CFTR cell surface expression is partly regulated by the COMMD1/CFTR interaction. We also show that COMMD1 is predominantly localized in the early/recycling endosomes and is absent from the ER and the trans-Golgi network. COMMD1 is located in the vesicles of the endocytic pathway as was previously observed in untransfected HeLa cells [12] and in HepG2 cells [29]. Our data expand on these studies and show that COMMD1 is located in early/recycling endosomes in the cytoplasm, whereas little protein is detected in the TGN. EHD1, also known as Rme-1, was initially described as a regulator of the TfR recycling from the endocytic recycling compartment to the plasma membrane [30]. Since then, EHD1 has been reported to regulate the recycling of a wide array of receptors, including CFTR [31]. We observed a partial colocalization between COMMD1 and these three proteins (CFTR, TfR, EHD1), suggesting that COMMD1 is involved in recycling. Indeed, COMMD1/CFTR colocalization was observed in a peripheral vesicular compartment. In addition, COMMD1 loss-of-function studies, in which CFTR cell surface expression was strongly reduced, showed a functional interaction between both proteins in such compartments. The presence of COMMD1 may influence the fate of CFTR by shifting the equilibrium from recycling and apical membrane expression to endocytosis. Internalized membrane proteins can be either targeted for degradation or recycled back to the plasma membrane, as demonstrated in a recent study where COMMD1 down-regulated ENaC trafficking [18].

Recently, COMMD1 was shown to bind with high specificity to an important signaling and regulatory lipid, phosphatidylinositol 4,5-bisphosphate (PIP_2_), and to be localized to the endosomal compartment, where it forms oligomers stabilized by interactions with lipids [29]. PIP_2_ anchors numerous signaling and cytoskeletal molecules to the cell membrane and plays various roles in membrane trafficking. Ion transporters and channels are regulated by PIP_2_. Phospholipid microdomains are thought to assemble PIP_2_ binding partners into signaling complexes and to control the activity of ion transporters and channels, such as ENaC [32] and CFTR [33], during biosynthesis or vesicle trafficking. We suggest that COMMD1 and CFTR could be recruited transiently in such domains, as could ENaC.

The pleiotropic functions of COMMD1 have been attributed to its role in the regulation of protein degradation [14]. This conclusion was based on results describing COMMD1 function within the copper homeostasis, HIF-1 and NF-κB pathways [11], [23]–[25]. Within the NF-κB pathway, COMMD1 specifically promotes the ubiquitination and subsequent nuclear proteasomal degradation of RelA, whereas it protects IκBα from the same outcome in the cytoplasm. Recently, COMMD1 was described as being associated with ENaC trafficking through ubiquitination of the channel at the cell surface and inhibition of channel recycling [18]. In this study, we show that down-regulation of COMMD1 increases ubiquitinated CFTR, whereas its overexpression decreases ubiquitinated CFTR. These experiments were performed without proteasome inhibitors to observe exclusively the effect of COMMD1 without altering overall protein stability and degradation. Enhanced stability due to impaired proteasomal degradation was described for IκBα, indicating that COMMD1 might have the same effect as a proteasome inhibitor, enhancing and sustaining phospho-IκB expression [23]. Our results reveal a similar mechanism by which COMMD1 selectively regulates CFTR trafficking through ubiquitination and leads to increased CFTR stability.

Critical residues within ICL3 have been identified, namely Ser-945 and Lys-946, through mutagenesis experiments that showed a loss of interaction between COMMD1 and the S945L-ICL3 mutant. COMMD1 overexpression clearly inhibited wt-CFTR ubiquitination, whereas it strongly induced ubiquitination of K946R- and K951R-CFTR. These results demonstrate that COMMD1 interacts through its COMM domain with the N-terminal end of ICL3 to modulate CFTR ubiquitination (Figure 4D). Thus, ICL3 is critical for COMMD1-mediated CFTR ubiquitination.

Ubiquitination is the major mode of regulation for the subcellular localization and turnover of member proteins. Ubiquitination of integral membrane proteins mediates their post-endocytic sorting to lysosomes, and a role for ubiquitination in the internalization of such proteins from the plasma membrane is emerging [34]. Recently, Ubiquitin Specific Protease-10 (USP10) has been shown to deubiquitinate CFTR in early endosomes and to enhance the endocytic recycling of CFTR [9]. We propose that COMMD1 might act as a scaffold in the endocytic compartment, recruiting enzymes required for the regulation of the ubiquitination of several membrane transporters. COMMD1 interacts with an E3 ubiquitin-ligase, X-linked inhibitor apoptosis (XIAP) [28], and a number of subunits of E3 ubiquitin-ligase complexes such as SCF^βTrCP^ [23] and ECS^SOCS1^ [11], [24], [35]. USP10 and a still unidentified E3 ligase could cooperate in such a scaffold to regulate the sorting of CFTR protein to the cell surface [9]. However, the temporal recruitment of these factors remains to be determined.

Regulation of the ubiquitin pathway was proposed to be the basis of many of the functions of the COMMD protein family contributing to protein degradation [13]. We suggest that COMMD1 might have a specific function within the endocytic machinery, where COMMD1-mediated ubiquitination might alter protein trafficking either by enhancing the cell surface expression of CFTR or by enhancing the internalization of ENaC.

A main feature of CF is the abnormal balance between fluid and electrolyte transport, leading to reduced chloride secretion with abnormal sodium absorption in the pulmonary epithelium [2]. A number of cellular functions have been attributed to CFTR, among which is the down-regulation of the transepithelial sodium transport mediated by ENaC [36]. Increasing COMMD1 expression may provide an approach to simultaneously inhibit ENaC absorption and enhance CFTR trafficking, two major issues underlying CF pathogenesis.

## Materials and Methods

### Antibodies

The following antibodies were used: MAB25031 (clone 24-1, R&D systems) and MM13-4 (Upstate), anti-CFTR monoclonal antibodies (mAb), anti-c-MYC mAb (9E10, BD Biosciences), anti-COMMD1 mouse mAb (2A12, Abnova) and rabbit pAb (Proteintech Group), mouse anti-Ub (P4D1, Santa Cruz Biotechnology), mouse anti-α-tubulin (12G10) and anti-α-subunit Na/K-ATPase α5 (DSHB, University of Iowa), goat anti-EHD1 (A20, Santa Cruz Biotechnology), mouse anti-TfR (clone H68.4, Zymed Laboratories), mouse anti-58k Golgi protein and mouse anti-calnexin (ab6284 and ab31290, Abcam), anti-Rab4 and -Rab11 mouse antibodies (BD Biosciences), Alexa Fluor 488 and 555-conjugated goat anti-mouse or anti-rabbit (Molecular Probes), horse radish peroxidase (HRP)-conjugated secondary antibodies (Pierce Biotechnology Inc) and HRP-conjugated secondary anti-IgG1 antibody (SouthernBiotech).

### Plasmids constructs and siRNA

The entire coding sequences of the human COMMD1 cDNA was isolated by PCR from a human brain cDNA library and subcloned into the plasmid pcDNA3.1/V5-His Topo (Invitrogen). Myc-COMMD1 and Myc-COMMD1ΔCOMM mutants were constructed using the Gene-Editor kit (Promega) and the resulting mutants were fully sequenced. CFTR plasmids were constructed in the expression vector pTracer-CMV (Invitrogen) as previously described [15]. COMMD1 siRNA sequence was 5′-GUCUAUUGCGUCUGCAGAC-3′ [23]. ON-TARGETplus SMARTpool of 4 siRNA was used. Control siRNA was siCONTROL Non-Targeting to demonstrate the absence of off-targets effects (Dharmacon Inc).

### Cell culture and transfection

HT-29 (ATCC, HTB-38), HeLa (ATCC, CCL-2) and HeLa cells stably transfected with wild-type CFTR (spTCF-wt) or pTracer (spTracer) were cultured and transfected as described [15].

### Immunofluorescent staining

Immunofluorescent staining was performed as previously described [15]. Cells were incubated overnight at 4°C with primary antibody (MAB25031 1∶200, rabbit pAb anti-COMMD1 1∶100, calnexin 1∶400, 58k Golgi 1∶100, Rab4 1∶100, Rab11 1∶100, EHD1 1∶100, TfR 1∶200). Control non-immune anti-mouse and anti-rabbit were used to remove non-specific signals. Fluorescence images of cells were captured and analyzed with a Leica DMR epifluorescence and a Zeiss Axio Observer Z.1 confocal microscope.

### Co-immunoprecipitation (CoIP)

Transfected cells were harvested in PBS1X, pelleted at 1000 g at 4°C and resuspended in 300 µl Co-IP buffer as previously described [15]. After preclearing, 70 µg of lysate was withdrawn for measurement of protein loading and the remaining material was incubated with 0.8 µg anti-CFTR (R&D Systems) or 0.8 µg anti-COMMD1 (Abnova) overnight at 4°C.

### Biotinylation and pull-down assays

Cells were washed three times in ice-cold PBS1X and then exposed to 0.25 mg/ml Sulfo-NHS-LC-biotin (Pierce Biotechnology Inc) in PBS1X for 30 minutes at 4°C. Cells were rinsed three times with PBS1X and then with glycine quenching buffer (Tris 50 mM, NaCl 125 mM, Glycine 190 mM, pH 7.4). Cells were harvested in glycine quenching buffer, pelleted at 1000 g at 4°C and lysed in 300 µl RIPA buffer. After preclearing, equal amounts of protein, as measured by the Bradford assay (Pierce Biotechnology Inc.), were used for enrichment of cell surface proteins by biotinylation and for loading controls (70 µg of lysate). The samples were incubated with streptavidin beads pre-equilibrated with RIPA buffer for 30 minutes at 4°C. The beads were washed four times with RIPA buffer. The biotinylation samples were subjected to SDS-PAGE and then analyzed by western blotting.

### Immunoprecipitation for ubiquitination assays

CFTR protein immunoprecipitation using MAB25031 was performed as previously described [15]. The totality of the immunoprecipitated sample was loaded into two halves on two lanes and subjected to SDS-PAGE 8% followed by western blotting analysis.

### CHX chase

In some experiments designed to monitor the kinetics of the mature CFTR protein, cells were incubated in the presence of 100 µg/ml cycloheximide (CHX) and input (70 µg of lysate) was subjected to SDS-PAGE 8% followed by western blotting analysis.

### Densitometry and statistical analysis

Western blot analysis and quantification was conducted on a Syngene BioImaging GeneGnome by lowest slope baseline correction. Data are presented as mean ± SE. Statistical significance was assessed by Student's *t*-test. Statistical significance was set at *P*<0.05; *n* indicates the number of independent experiments.
